# Aortic annulus sizing in bicuspid and tricuspid aortic valves using CT in patients with surgical aortic valve replacement

**DOI:** 10.1038/s41598-021-00406-3

**Published:** 2021-10-25

**Authors:** Jooae Choe, Hyun Jung Koo, Joon-Won Kang, Joon Bum Kim, Hee Jun Kang, Dong Hyun Yang

**Affiliations:** 1 Department of Radiology and Research Institute of Radiology, Cardiac Imaging Center, Asan Medical Center, University of Ulsan College of Medicine, Olympic-ro 43 gil, 88, Song-pa gu, Seoul, 05505 South Korea; 2 Cardiovascular Surgery, Asan Medical Center, University of Ulsan College of Medicine, Seoul, South Korea; 3 Cardiology, Asan Medical Center, University of Ulsan College of Medicine, Seoul, South Korea

**Keywords:** Cardiology, Medical research, Outcomes research

## Abstract

The purpose of this study was to evaluate whether bicuspid anatomy affects the discrepancy between CT-derived annular size and intraoperative size. We retrospectively analyzed annular measurements in 667 patients who underwent surgical aortic valve replacement (AVR). Preoperative CT measurements of the aortic annulus were compared to surgically implanted valve sizes. To evaluate whether the bicuspid valve affects the differences between CT annulus diameter and surgical AVR size, patients with diameter larger by > 10% (CT-Lg group) on CT, compared to surgical AVR size, were compared with those having size difference < 10% (CT-Sim group). Propensity score matching yielded 183 matched patients from each group. Bicuspid aortic valve annulus parameters significantly correlated with surgical aortic valve size (r = 0.52–0.71; for all, *p* < 0.01). The most representative measurements corresponded to surgical aortic valve size were area-derived diameters in tricuspid aortic valve (r = 0.69, *p* < 0.001) and bicuspid without raphe (r = 0.71, *p* < 0.001), and perimeter-derived diameter in bicuspid with raphe (r = 0.63, *p* < 0.001). After propensity score matching, native valve type was not different between CT-Sim and CT-Lg groups. In multivariable analysis, the difference between CT-derived diameter and surgical AVR size was affected by the operator factor and types of prosthesis. Bicuspid aortic annulus diameters measured on CT showed a significant correlation with surgical aortic valve size. The difference between CT-derived diameter and surgical AVR size is affected by operator factor and the types of prosthesis but not affected by the bicuspid valve.

## Introduction

Surgical aortic valve replacement (SAVR) has been the main option for symptomatic aortic valvular diseases. Over the last decade, transcatheter aortic valve replacement (TAVR) has been rapidly accepted as an alternative for inoperable patients or those at high perioperative risk. Aortic valvular sizing is important for both procedures, to avoid paravalvular leakage, annular rupture, ostial coronary occlusion, and other complications. Moderate paravalvular leakage is reportedly rare (3.5%) at 30 days after TAVR but increases the risk of death and heart failure a year later^1^. The selection of an oversizing valve that fits the annulus plane is important to prevent paravalvular leakage, prosthetic valve migration, or prosthetic-patient mismatch, which can affect left ventricular mass regression and subsequent survival^2–4^. Nowadays, computed tomography (CT) has been considered a procedure of choice for aortic annular sizing, including the detection of coronal ostial location and aortic root anatomy^2,5^.

Bicuspid aortic valve is characterized by the anatomy of heavy calcium deposition of leaflets, fibrotic or calcific raphes, commissural fusions, more elliptical orfices and it is also associated with a greater dilatation of ascending thoracic aorta dimensions compared with tricuspid aortic valve^6^. The morphologic finding (presence of raphe) in bicuspid aortic valve was associated with poor prognostic outcome with increased rates of valvular dysfunction, aortic valve and aorta surgery and also the outcomes of TAVR in bicuspid aortic stenosis depended on valve morphology^7,8^. Therefore, morphologic evaluation of bicuspid aortic valve is needed before both SAVR or TAVR.

Although the valve sizing is based on the manufacturer-specific valve sizer during the SAVR, TAVR sizing is based on transesophageal echocardiography and electrocardiogram (ECG)-gated multidetector computed tomographic imaging. Therefore, the size measurement is especially important for patients candidate for TAVR. Aortic annular measurement methods using CT have been evaluated using diverse approaches involving the annulus diameter, area, or perimeters in the tricuspid aortic valve anatomy^9,10^; however, the bicuspid aortic valve was excluded in previous studies. Bicuspid aortic valve represents a challenge for an adequate sizing of prosthesis due to its anatomical distinctiveness. The three aortic valve leaflets of the tricuspid valve form a virtual ring at the basal attachments of the left ventricular outflow tract^11^, while the bicuspid valve has only two leaflets that cannot form a ring but form a line by connecting the two basal attachment sites making difficult to determine the annulus plane. However, we can visually select the smallest area, including the two basal attachment sites of bicuspid leaflets, similar to the tricuspid valve measurement method. Using the similar method, TAVR planning has been performed, and recent studies showed similar favorable outcomes of TAVR in patients with bicuspid aortic stenosis (AS)^12–14^. The outcomes of TAVR in bicuspid AS are suboptimal when using the first-generation transcatheter valves; however, the newer-generation transcatheter valves showed improved outcomes of TAVR^12–14^. Considering the expanding indications of TAVR in bicuspid aortic valve, aortic annular sizing in bicuspid valves should be evaluated.

Therefore, we hypothesized that aortic annular sizing on preoperative systolic phase (20–30% of R-R interval) CT correlates with the intraoperative valve sizing for SVAR in both bicuspid and tricuspid aortic valves. To evaluate whether the bicuspid anatomy affects the discrepancy between CT-derived annular size and intraoperative size, we compared patients with a difference of > 10% and ≤ 10% between the sizes measured by CT and those measured at surgery.

## Methods

### Patients

This study was approved by the Institutional Review Board of Asan Medical Center (approval number: 2018-0233) and informed consent requirement was waived. A registry-based retrospective consecutive cohort study was conducted with patients who underwent SVAR with a tissue or mechanical valve and had preoperative MDCT scan between June 2011 and March 2016. Exclusion criteria included active infection, cardiogenic shock, and concurrent intramural hematoma or dissection. Additional exclusion criteria were patients with no multiphase CT data (n = 34), concurrent aorta wrapping (n = 30) or annular widening (n = 11) operations, quadricuspid aortic valves (n = 3) and sinus of Valsalva aneurysm rupture (n = 1). A total of 667 patients (362 tricuspids, 166 bicuspids with raphe, and 139 bicuspids without raphe) were included in this study. Clinical information and echocardiographic findings were collected using electronic medical records. Preoperative and postoperative transthoracic echocardiograms were routinely performed. All methods were performed in accordance with the relevant guidelines and regulations.

### CT protocol and aortic root measurement on CT

**Figure 1:**
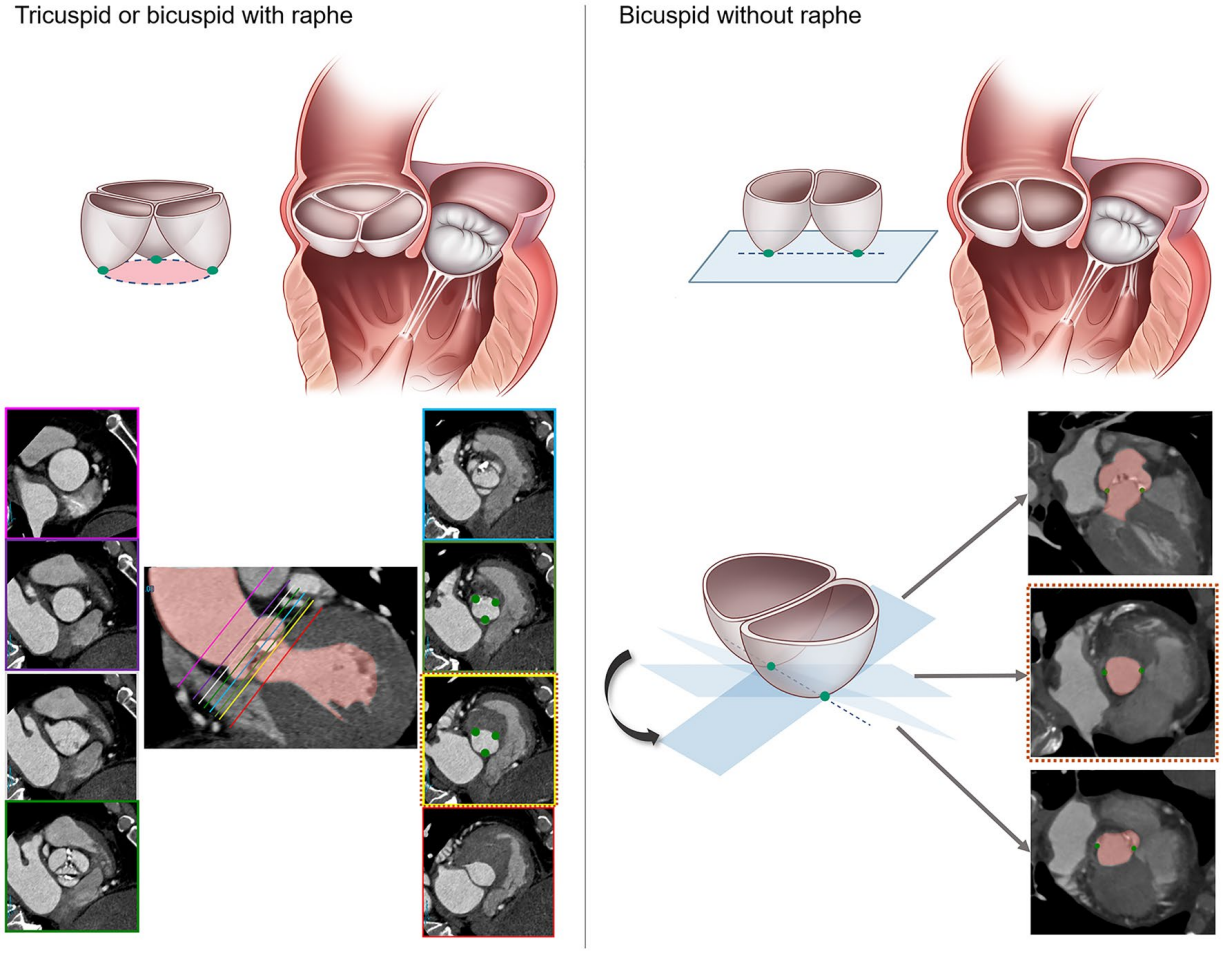
Measurement of virtual ring of aortic annulus on CT. In tricuspid or bicuspid aortic valve with raphe, descending from the sinus Valsalva to left ventricular outflow track, the hinge point of each individual aortic valve cusp is identified to establish the plane of the annulus. Virtual ring of aortic annulus is selected at the plane of the circumferential ring at the basal attachment points of the three aortic cusps. In bicuspid valve without raphe, the plane and level of virtual ring of aortic annulus is determined when the smallest cross-sectional area is drawn, by drawing a line between both basal ends of the cusps and turning the plane 360 around those two points.

Cardiac CT scanning was performed using dual-source CT scanners (Somatom Force or Somatom Definition Flash, Siemens, Erlangen, Germany) with retrospective electrocardiogram-gating. Detailed CT protocol is described in Supplementary material. Aortic root evaluation methods were described in previous articles^15–18^. End-systolic phase (20–30% of R-R interval) CT images were used to find the optimal CT-derived annular diameter^9^. Evaluation of CT images was independently performed by an experienced cardiac radiologist blinded to echocardiographic and clinical information. To assess interobserver agreement of CT measurements, another experienced cardiac radiologist measured CT-derived annular parameters of 100 randomly selected patients. The aortic annular level was selected at the plane of the circumferential ring at basal attachment points of three aortic cusps for tricuspid and bicuspid with raphe. In bicuspid without raphe, the plane and level of annulus were determined when the smallest cross-sectional area was drawn, taking both basal ends of the cusp and turning the plane 360 degrees around those two points (Fig. 1). Manually drawn minimal and maximal annular diameters, mean diameters, perimeter-derived diameters, and area-derived diameters at the aortic annular level were obtained. If calcified plaques existed at the aortic annular level, the annular parameters were obtained based on the assumption that there were no calcified plaques, and the area of calcified plaques at the aortic annular level was additionally measured. The maximal diameters of aortic sinus, sinotubular junction, and ascending aorta tubular portion were also obtained.

### Surgical aortic valve replacement

Operation procedures were performed based on the surgeons’ preference (five expert cardiac surgeons), mainly using a standard median sternotomy approach. After resection of the aortic valve cusps and debridement of calcifications, intraoperative aortic annulus sizing was performed using a manufacturers’ sizer to define an optimal valve size. A specific valve sizer was used for each prosthetic valve type, as indicated. No patient underwent aortic root reduction or ascending aorta replacement procedures.

### Valve sizing scales in implanted valve and CT-based measurements

Nominal valve sizes from different manufacturers have unequal inner diameters, and the surfaces of the valve touching the annulus are different tissue planes. Therefore, as a reference standard for the intraoperative annulus sizing, we used the ‘tissue annulus diameters (TAD)’ according to the different valve types where the valve and the annulus met, rather than the nominal valve size^19^.

In this study, we assessed the correlation between CT-derived diameters and TAD of surgically implanted valves, and area-derived annular diameters measured on systolic phase CT were used as a representative value of CT annular sizing. Percentage differences between CT-derived annular diameter and TAD were defined as follows:

$$\% \,{\text{Difference }} = \, \left( {{\text{Area-derived}}\,{\text{annular}}\,{\text{diameter}}\,{\text{on}}\,{\text{CT }}{-}{\text{ TAD}}} \right)/{\text{TAD}} \times 100$$

Primarily, we classified patients into two groups, CT-Sim: %Difference ≤ 10, and CT-Lg: %Difference > 10 (Supplementary Table 1) and compared between two groups to evaluate associated factors that affect the difference between CT-derived annular diameter and surgical TAD.

### Comparison of geometric orifice area

The size and geometric orifice area (GOA), calculated from the actual inner diameter of each implanted valve brand, were also obtained, to quantify the difference of intraoperative valve size versus CT-based sizing: GOA = (inner diameter/2)^2^ × π. The details of theoretical SAVR valve size according to the CT-based measurement was determined based on the following scale:

- Area-derived diameter on CT 19.0–20.9 mm: valve size 19, GOA, 2.270 cm^2^
- Area-derived diameter on CT 21.0–22.9 mm: valve size 21, GOA, 2.835 cm^2^
- Area-derived diameter on CT 23.0–24.9 mm: valve size 23, GOA, 3.464 cm^2^
- Area-derived diameter on CT 25.0–26.9 mm: valve size 25, GOA 4.155 cm^2^
- Area-derived diameter on CT 27.0–28.9 mm: valve size 27, GOA, 4.909 cm^2^
- Area-derived diameter on CT ≥ 29.0 mm: valve size 29, GOA, 5.726 cm^2^

GOA of the theoretical SAVR valves was based on Carpentier Edward Magna valves, the most commonly (32.3%) used valve in this study. Additional analysis of GOA values (area-derived GOA) when the same patient received TAVR using SAPIEN 3 (Edwards Lifesciences, Irvine, CA) was included as a theoretical comparison in Supplementary Figure 1.

### Statistical analysis

Demographic and laboratory data were presented as median and interquartile ranges for continuous variables, and numbers of subjects (percentages) for categorical variables. Patient characteristics were compared between groups using the Student *t*-test or chi-square test, as appropriate, according to data types. To evaluate reliability of CT measurement, inter-observer agreement was obtained using a two-way random model intra-class correlation coefficient (ICC). Correlation between surgical AV size (TAD) and CT-derived diameters of three types of native aortic valves were demonstrated by Pearson correlation methods. A box plot was created to compare implanted valve size and area-derived annulus diameter. To evaluate associated factors that affects the difference between CT-derived annular diameter and TAD, we compared between patients with diameter larger by ˃ 10% on CT than the surgical AVR size (group CT-Lg) and those with difference ≤ 10% (group CT-Sim) using univariable analysis and multivariable analyses after propensity score matching. Age, sex, atrial fibrillation, AS dominancy, cerebrovascular accident, hypertension, heart failure, liver cirrhosis, creatinine, brain natriuretic peptide (BNP), ejection fraction (EF), left ventricular mass index (LVMI), peak velocity, and prosthetic valve types and operators were used as covariates for propensity score matching with automated balance optimization. To identify significant factors that affect the discrepancy between CT-derived diameter and intraoperative sizing, all clinically relevant variables with *p* < 0.1 in the univariate analysis were subjected to a multivariable logistic regression analysis. A *p* < 0.05 was considered statistically significant. Statistical analyses were performed with SAS version 9.3 (SAS Institute, Cary, NC) or SPSS software version 20.0 (IBM Corp., Armonk, NY, USA).

## Results

### Baseline characteristics

Baseline demographics and clinical characteristics were compared among the patients with tricuspid (n = 362), bicuspid without raphe (n = 139) and bicuspid with raphe (n = 166) (Table 1). Patients with bicuspid valves were younger than those with tricuspid aortic valves (68 years vs. 62 years). Bicuspid valves with raphe were predominant in males (76%). Mean nominal valve size of implanted valves was larger in bicuspid with raphe (23.4 vs. 22.3 mm, *p* < 0.001). TAD was smaller compared to CT-derived sizing, regardless of tricuspid or bicuspid shape (tricuspid valve, TAD 22.3 vs. area-derived annulus diameter 25.2 mm). Annular diameters (maximal, minimal, perimeter-derived and area-derived) were larger in patients with bicuspid valve with raphe compared to those with tricuspid valve (for all comparison, *p* < 0.001). Calcification burden at the annular level was larger in bicuspid valves compared to tricuspid valves (9.0 mm^2^ in bicuspid valves without raphe vs. 2.9 mm^2^, *p* = 0.001). Sinus diameter did not show statistical difference among the three groups. ST junction diameter and ascending aorta tubular portion diameter were larger in bicuspid valves with/without raphe compared to those in tricuspid aortic valves.

**Table 1:** Clinical characteristics and echocardiographic findings.

Variables	Tricuspid (n = 362)	Bicuspid without raphe (n = 139)	Bicuspid with raphe (n = 166)	*p* value*	*p* value^†^
Age, year	67.9 ± 10.1	61.7 ± 9.0	62.0 ± 11.7	< 0.001	< 0.001
Sex, male	211 (58.3)	69 (49.6)	126 (75.9)	0.08	< 0.001
Prosthetic valve types				< 0.001	< 0.001
Hancock	71 (19.6)	16 (11.5)	26 (15.7)		
SJR	56 (15.5)	46 (33.1)	41 (24.7)		
ATS AP	56 (15.5)	29 (20.9)	40 (24.1)		
CE magna	144 (39.8)	31 (22.3)	41 (24.7)		
Others‡	35 (9.7)	17 (12.2)	18 (10.8)		
Operator				0.63	0.51
Operator 1	66 (18.2)	29 (20.9)	35 (21.1)		
Operator 2	136 (37.6)	45 (32.4)	66 (39.8)		
Operator 3	35 (9.7)	10 (7.2)	12 (7.2)		
Operator 4	55 (15.2)	23 (16.5)	29 (17.5)		
Operator 5	70 (19.4)	32 (23.0)	24 (14.5)		
Mitral regurgitation	37 (10.2)	7 (5.0)	12 (7.2)	0.07	0.14
Atrial fibrillation	63 (17.4)	16 (11.5)	31 (18.7)	0.11	0.19
AS dominancy	249 (68.8)	130 (93.5)	124 (74.7)	< 0.001	< 0.001
Pure AS	169 (46.7)	105 (75.5)	90 (54.2)	< 0.001	0.11
ASr	80 (22.1)	25 (18.0)	34 (20.5)	0.31	0.68
CVA	71 (19.6)	16 (11.5)	18 (10.8)	0.03	0.01
Hypertension	137 (37.8)	76 (54.7)	97 (58.4)	0.001	< 0.001
Peripheral arterial disease	1 (0.3)	1 (0.7)	2 (1.2)	0.48	0.43
Rheumatic valvular disease	50 (13.8)	6 (0.4)	14 (8.4)	0.003	0.005
COPD	15 (4.1)	1 (0.7)	7 (4.2)	0.05	0.14
Diabetes mellitus	85 (23.5)	20 (14.4)	41 (24.7)	0.03	0.053
Heart failure	43 (11.9)	5 (3.6)	12 (7.2)	0.004	0.01
History of malignancy	35 (9.7)	15 (9.0)	13 (7.8)	0.71	0.66
Dyslipidemia	39 (10.8)	15 (9.0)	14 (8.4)	1.00	0.69
History of myocardial infarct	6 (1.7)	1 (0.7)	4 (2.4)	0.68	0.51
Liver cirrhosis	3 (0.8)	2 (1.2)	3 (1.8)	0.62	0.61
Chronic renal failure	26 (7.2)	2 (1.2)	7 (4.2)	0.009	0.028
Albumin, g/dL	3.7 ± 0.4	3.8 ± 0.4	3.8 ± 0.4	< 0.001	< 0.001
BUN, mg/dL	19.8 ± 9.5	16.4 ± 5.5	17.9 ± 6.4	< 0.001	0.033
Creatinine, mg/dL	12.7 ± 1.7	13.1 ± 1.6	13.6 ± 1.7	0.002	0.135
BNP, pg/dL	138 ± 336.5	80 ± 207	87 ± 202.8	0.005	0.006
Hemoglobin, g/dL	12.7 ± 1.7	13.1 ± 1.6	13.6 ± 1.7	0.014	< 0.001
Preoperative echocardiography					
LVEF, %	56.3 ± 12.5	60.1 ± 10.5	56.4 ± 11.7	0.004	0.99
LVMI, g/m^2^	148.3 ± 46.0	138.6 ± 38.3	150.8 ± 47.1	0.078	0.82
Peak velocity, m/s	4.3 ± 1.4	4.9 ± 1.2	4.4 ± 1.3	< 0.001	0.72
Mean pressure gradient, mmHg	56.5 ± 22.8	64.8 ± 23.1	55.5 ± 25.6	0.003	0.90
Postoperative echocardiography					
LVEF, %	54.5 ± 12.8	60.7 ± 8.6	54.6 ± 13.6	0.004	0.99
LVMI, g/m^2^	218.1 ± 210.0	190.1 ± 63.8	213.3 ± 77.3	0.08	0.82
Peak velocity, m/s	2.7 ± 0.6	2.6 ± 0.5	2.5 ± 0.6	< 0.001	0.72
Mean pressure gradient, mmHg	16.1 ± 7.0	14.8 ± 5.8	14.7 ± 6.0	0.003	0.903

Data are shown as the number of patients with percentages in parentheses or mean ± standard deviation.

AS, aortic stenosis; ASr, aortic stenosis and minimal to mild aortic regurgitation; BNP, brain natriuretic peptide; BUN, blood urea nitrogen; CE magna, Carpentier-Edwards PERIMOUNT Magna; COPD, chronic obstructive pulmonary diseases; CVA, cerebrovascular accident; LVEF, left ventricular ejection fraction; LVMI, left ventricular mass index; SJR, St Jude Medical Regent.

*p values are obtained by comparing between tricuspid and bicuspid without raphe and ^†^between tricuspid and bicuspid with raphe. ^‡^Valves including Sorin (n = 24), On-X (n = 12), CM TopHat (n = 11), Trifecta (n = 11), Mitroflow (n = 8), Mosaic (n = 3) and Biocor (n = 1).

Among all patients who underwent SAVR, 75% (503/667) had aortic stenosis, and the percentages of patients with aortic stenosis were higher in bicuspid valve groups. On preoperative echocardiography, patients who had bicuspid valves without raphe presented higher peak velocity and mean transvalvular pressure gradient among the three groups. Heart failure was noted in 12% (43/362) of patients with tricuspid valves, and BNP was higher in this group compared to bicuspid with/without raphe groups. After the operation, peak velocity and mean pressure gradient were normalized in the three groups. The percentage of patient-prosthesis mismatch was not statistically different among the groups.

### Optimal CT-derived annular diameter

Inter-observer agreement of aortic root measurements on CT was good (ICC: 89.2–98.6) (Supplementary Table 2). The Pearson correlation between CT-derived annular diameters and intraoperative valve sizes are noted in Table 3. Bicuspid aortic valve parameters were significantly correlated with surgical aortic valve sizes as well as tricuspid valve parameters (*r* = 0.52–0.71). Of all measurements, area-derived dimeters showed the strongest correlation with tissue annular diameter of surgical valves in patients with tricuspid (r = 0.69, *p* < 0.001) or bicuspid without raphe (*r* = 0.71, *p* < 0.001). In patients with bicuspid with raphe, perimeter-derived diameter was the strongest parameter and demonstrated the highest correlation (*r* = 0.63, *p* < 0.001). Based on the correlation results, we selected area-derived diameters to compare the discrepancy between CT-derived diameters and tissue annular diameters of surgical valves.

### Comparison of CT vs. Intraoperative valve sizing

**Figure 2:**
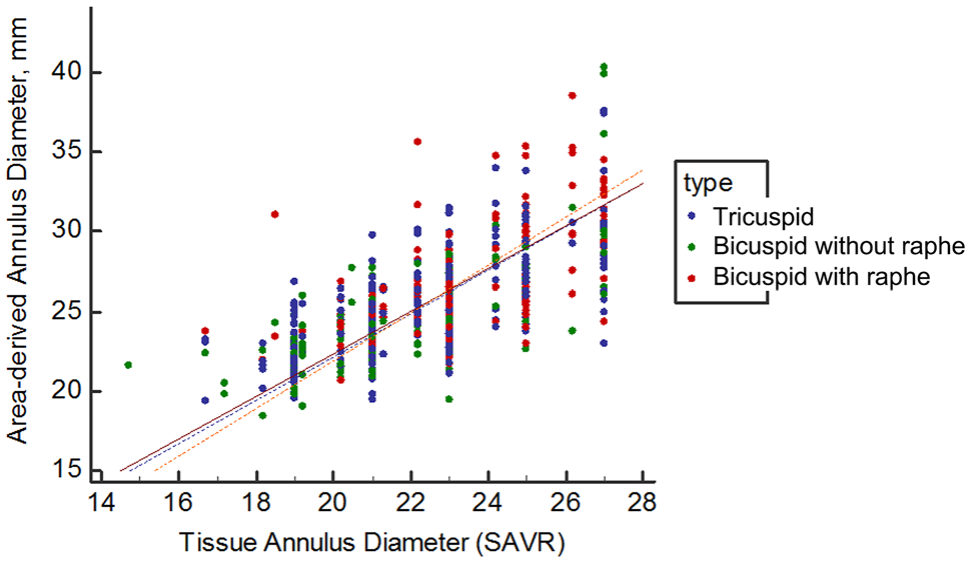
Scatter plot to demonstrate the distribution of area-derived annular diameter measured on CT and tissue annular diameter of surgical aortic valve replacement (SAVR).

**Figure 3:**
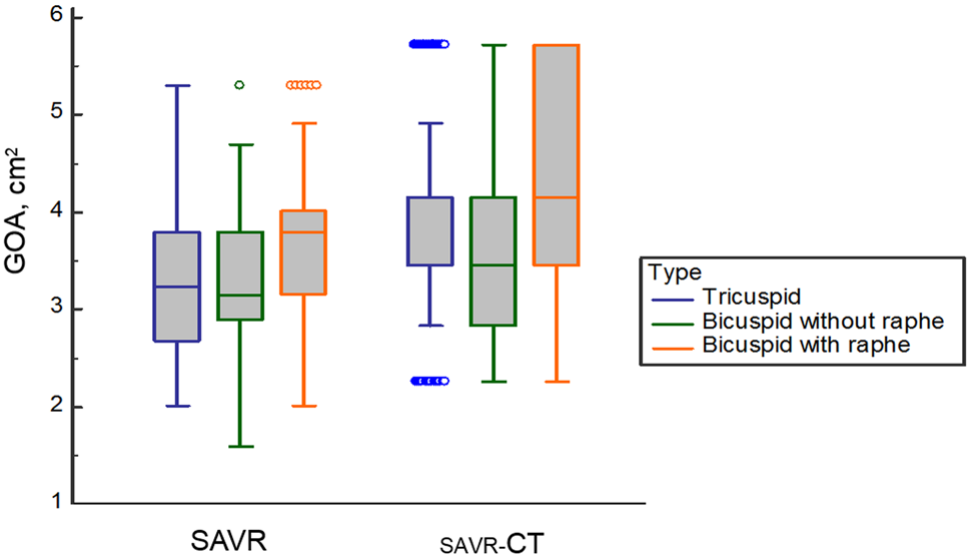
Comparison of GOA measured in surgical aortic valve replacement (SAVR) and CT-derived geometric orifice area (_SAVR_-CT) according to the native valve types. GOA, geometric orifice area; SAVR, surgical aortic valve replacement.

Figure 2 shows a scatter plot demonstrating the distribution of TAD and area-derived annular diameter. Box plot to demonstrate the nominated implanted valve size and area-derived annular diameter was presented in Supplementary figure 2. Intraoperative sizing in SAVR was smaller than CT-derived sizing regardless of tricuspid or bicuspid valve shape (Fig. 3). Intraoperative GOA, area-derived diameter on CT, and suggested surgical valve size based on CT were not statistically different between tricuspid and bicuspid valve without raphe groups (*p* > 0.05; Table 2). However, the values were significantly larger in the bicuspid valve with raphe group compared to tricuspid group (SAVR-CT GOA, 3.9 cm^2^ vs. 4.4 cm^2^, *p* < 0.001; Fig. 3).

**Table 2:** Surgical aortic valve size and CT measurements.

Variables	Tricuspid (n = 362)	Bicuspid without raphe (n = 139)	Bicuspid with raphe (n = 166)	*p* value*	*p* value^†^
Implanted valve size, mm	22.3 ± 2.2	22.1 ± 2.3	23.4 ± 2.2	0.73	< 0.001
Tissue annulus diameter, mm	22.3 ± 2.3	22.0 ± 2.5	23.4 ± 2.3	0.34	< 0.001
Intraoperative sizing					
GOA, cm^2^	3.4 ± 0.7	3.3 ± 0.7	3.7 ± 0.7	0.17	< 0.001
Indexed GOA	2.0 ± 0.4	1.9 ± 0.4	2.1 ± 0.4	0.20	0.001
CT-based SAVR sizing					
Area-derived diameter, mm	25.2 ± 3.1	24.9 ± 3.4	27.0 ± 3.4	0.34	< 0.001
SAVR-CT GOA, cm^2^	3.9 ± 1.0	3.76 ± 1.0	4.4 ± 1.0	0.20	< 0.001
Indexed SAVR-CT GOA, cm^2^	2.4 ± 0.5	2.3 ± 0.5	2.6 ± 0.5	0.21	< 0.001
GOA_CT _− GOA_SAVR_, cm^2^	1.2 ± 0.2	1.2 ± 0.2	1.2 ± 0.2	0.93	0.19
CT-derived parameters					
Maximal annulus diameter, mm	28.3 ± 3.5	27.3 ± 4.1	30.0 ± 4.0	0.03	< 0.001
Minimal annulus diameter, mm	22.9 ± 3.1	22.8 ± 3.2	24.4 ± 3.6	0.99	< 0.001
Mean annulus diameter, mm	25.6 ± 3.1	25.0 ± 3.4	27.2 ± 3.6	0.67	< 0.001
Annulus perimeter, mm	81.1 ± 10.1	80.2 ± 11.0	87.0 ± 11.1	0.73	< 0.001
Perimeter-derived annulus diameter, mm	25.8 ± 3.2	25.5 ± 3.5	27.7 ± 3.5	0.66	< 0.001
Annulus area, mm^2^	507.1 ± 130.0	496.5 ± 147.1	581.7 ± 152.9	0.73	< 0.001
Area-derived annulus diameter, mm	25.2 ± 3.1	24.9 ± 3.4	27.0 ± 3.4	0.61	< 0.001
Area of calcification at the annulus, mm^2^	2.9 ± 9.1	9.0 ± 26.6	7.4 ± 20.3	0.001	0.02
Sinus diameter, mm	38.6 ± 34.0	38.4 ± 5.2	39.4 ± 4.6	0.99	0.95
Sinotubular junction diameter, mm	31.0 ± 4.8	32.9 ± 5.5	33.7 ± 5.4	< 0.001	< 0.001
Ascending aorta tubular portion, mm	38.3 ± 5.7	44.0 ± 6.3	42.5 ± 6.5	< 0.001	< 0.001
Patient-prosthesis mismatch^‡^				0.13	0.17
Normal	279 (77.1)	116 (83.5)	137 (82.3)		
Mild/moderate	81 (22.4)	23 (16.5)	29 (17.5)		
Severe	1 (0.3)	0 (0)	0 (0)		

Data are shown as the number of patients with percentages in parentheses or mean ± standard deviation.

GOA, geometric orifice area; SAVR, surgical aortic valve replacement.

*p values are obtained by comparing between tricuspid and bicuspid without raphe and ^†^between tricuspid and bicuspid with raphe. ^‡^*p* values are obtained by comparing between normal group and the group with mild to severe grade of patient-prosthesis mismatch.

A comparison was performed between patients with diameter larger by ˃ 10% on CT than the surgical AVR size (group CT-Lg) and those with difference ≤ 10% (group CT-Sim) (Table 3). Among the 667 patients, 244 were classified in group CT-Sim, and in group CT-Lg. Baseline demographics and comorbidities between the two groups were not similar (Table 4). Prosthetic valve types and operators were statistically different, and male dominancy was noted in group CT-Lg. In group CT-Lg, patients were younger and they presented with smaller EF, and had large LV mass index. Operator factor and prosthetic valve types were significantly different between the two groups. Bicuspid valve was not a significant factor affecting the discrepancy between CT and surgical sizing. Propensity score matching yielded 183 matched patients from each group. In the matched group, operator factor and prosthetic valve types were remained factors still affecting the discrepancy between CT-Sim and CT-Lg after multivariable logistic regression analysis (Table 5). Bicuspid valve was not a significant factor even after propensity score matching.

**Table 3:** Correlation between surgical aortic valve size (tissue-annulus diameter) and CT-derived annulus diameters of three types of native aortic valves (*r*, all data are *p* < 0.01).

CT-derived parameter	Tricuspid	Bicuspid with Raphe	Bicuspid without raphe
Maximal annulus diameter, mm	0.66	0.62	0.64
Minimal annulus diameter, mm	0.63	0.52	0.63
Mean annulus diameter, mm	0.68	0.61	0.68
Area-derived diameter, mm	0.69	0.62	0.71
Perimeter-derived diameter, mm	0.65	0.63	0.70

**Table 4:** Comparison between patients with > 10% larger diameter on CT from the surgical aortic valve size (CT-Lg, n = 423) and those ≤ 10% range (CT-Sim, n = 244), before and after applying propensity scores.

	Before propensity score matching				After propensity score matching			
	CT-Sim (n = 244)	CT-Lg (n = 423)	*p* value	SD	CT-Sim (n = 183)	CT-Lg (n = 183)	*p* value	SD
Prosthetic valve			< 0.001				0.20	
Hancock	71 (29.1)	42 (9.9)		− 0.50	52 (28.4)	36 (19.7)		− 0.21
SJR	40 (16.4)	103 (24.3)		0.20	28 (15.3)	34 (18.6)		0.09
ATS AP	30 (12.3)	95 (22.5)		0.27	23 (12.6)	35 (19.1)		0.18
CE magna	88 (36.1)	128 (30.3)		− 0.12	69 (37.7)	67 (36.6)		− 0.02
Others*	15 (6.1)	55 (13.0)		0.24	11 (6.0)	11 (6.0)		< 0.001
Operator			< 0.001				0.01	
Operator 1	85 (34.8)	45 (10.6)		− 0.60	53 (29.0)	27 (14.8)		− 0.35
Operator 2	79 (32.4)	168 (39.7)		0.15	67 (36.6)	81 (44.3)		0.16
Operator 3	25 (10.2)	32 (7.6)		− 0.09	20 (10.9)	19 (10.4)		− 0.02
Operator 4	34 (13.9)	73 (17.3)		0.09	25 (13.7)	25 (13.7)		< 0.001
Operator 5	21 (8.6)	105 (24.8)		0.44	18 (9.8)	31 (16.9)		0.21
Native valve shape			0.77				0.78	
Tricuspid	136 (55.7)	226 (53.5)		− 0.04	100 (54.6)	99 (54.1)		− 0.01
Bicuspid without raphe	51 (20.9)	88 (20.8)		− 0.002	39 (21.3)	35 (19.1)		− 0.05
Bicuspid with raphe	57 (23.4)	109 (25.8)		0.06	44 (24.0)	49 (26.8)		0.06
Age, year	68.2 ± 9.0	63.4 ± 11.2	< 0.001	− 0.48	68.9 ± 7.8	68.0 ± 8.9	0.28/0.26^†^	− 0.11
Sex, male	130 (53.3)	276 (65.2)	0.003	− 0.24	100 (54.6)	108 (59.0)	0.46	0.09
Atrial fibrillation	47 (19.3)	63 (14.9)	0.18	− 0.12	38 (20.8)	39 (21.3)	1.00	0.01
AS predominant	200 (82.0)	303 (71.6)	0.004	− 0.25	159 (86.9)	158 (86.3)	1.00	− 0.02
AR predominant	36 (14.8)	92 (21.7)	0.04	0.18	19 (10.4)	16 (8.7)	0.72	− 0.06
Mitral regurgitation	23 (9.4)	33 (7.8)	0.56	− 0.06	12 (6.6)	8 (4.4)	0.49	− 0.10
CVA	45 (18.4)	60 (14.2)	0.18	− 0.11	36 (19.7)	37 (20.2)	1.00	0.01
Hypertension	139 (57.0)	218 (51.5)	0.20	− 0.11	103 (56.3)	101 (55.2)	0.92	− 0.02
PAD	2 (0.8)	2 (0.5)	0.97	− 0.04	2 (1.1)	1 (0.5)	1.00	− 0.06
Rheumatic valve	23 (9.4)	47 (11.1)	0.58	0.06	19 (10.4)	14 (7.7)	0.47	− 0.10
COPD	8 (3.3)	15 (3.5)	1.00	0.01	5 (2.7)	6 (3.3)	1.00	0.03
Diabetes mellitus	50 (20.5)	96 (22.7)	0.57	0.05	44 (24.0)	49 (26.8)	0.63	0.06
Heart failure	16 (6.6)	44 (10.4)	0.13	− 0.32	11 (6.0)	11 (6.0)	1.00	< 0.001
Malignancy	19 (7.8)	44 (10.4)	0.33	0.09	17 (9.3)	26 (14.2)	0.19	0.15
Dyslipidemia	24 (9.8)	44 (10.4)	0.92	0.02	21 (11.5)	19 (10.4)	0.87	− 0.04
Chronic renal failure	10 (4.1)	25 (5.9)	0.41	0.08	8 (4.4)	10 (5.5)	0.81	0.05
History of MI	4 (1.6)	7 (1.7)	1.00	0.01	4 (2.2)	2 (1.1)	0.68	− 0.09
Liver cirrhosis	5 (2.0)	3 (0.7)	0.25	− 0.11	4 (2.2)	1 (0.5)	0.37	− 0.14
LVEF, %	58.6 ± 11.7	56.2 ± 12.1	0.01/0.001^†^	− 0.20	59.4 ± 11.0	58.7 ± 10.1	0.56/0.07^†^	− 0.06
LVMI, g/m^2^	142.1 ± 43.4	149.7 ± 45.6	0.04/0.03^†^	0.17	140.6 ± 43.2	141.8 ± 43.9	0.80/0.74^†^	0.03
Peak velocity, m/s	4.5 ± 1.2	4.4 ± 1.4	0.13/0.21^†^	− 0.12	4.8 ± 0.9	4.8 ± 1.0	0.94/0.87^†^	0.01
Mean PG, mmHg	58.0 ± 22.4	58.1 ± 24.7	0.98/0.99^†^	0.002	58.3 ± 22.1	58.3 ± 24.0	0.99/0.95^†^	0.001

Data are shown as the number of patients with percentages in parentheses or mean ± standard deviation unless specified otherwise. When the continuous variables do not follow the normal distribution, *t*-test result assuming the normality and the Shapiro–Wilk Test indicating the non-normal test are specified in the *p*-value (*p*-value in t-test/*p*-value in Shapiro–Wilk test)^†^. *Valves including Sorin (n = 24), On-X (n = 12), CM TopHat (n = 11), Trifecta (n = 11), Mitroflow (n = 8), Mosaic (n = 3) and Biocor (n = 1).

AR, aortic regurgitation; AS, aortic stenosis; CE magna, Carpentier-Edwards PERIMOUNT Magna; COPD, chronic obstructive pulmonary diseases; CVA, cerebrovascular accident; LVEF, left ventricular ejection fraction; LVMI, left ventricular mass index; MI, myocardial infarction; PAD, peripheral arterial disease; PG, pressure gradient; SD, standard deviation; SJR, St Jude Medical Regent.

**Table 5:** Univariable and multivariable logistic regression analysis after propensity score matching to discriminate the patients with > 10% larger area-derived annulus diameter on CT (CT-Lg) compared to tissue annulus diameter of prosthetic valve from those ≤ 10% range of differences (CT-Sim).

Variable	Univariable			Multivariable				
	$\mathrm{B}$	OR	95% CI	*p* value	$\mathrm{B}$	OR	95% CI	*p* value
Age	− 0.01	0.99	[0.96, 1.01]	0.28				
Sex	− 0.18	0.84	[0.55, 1.27]	0.40				
Operator 1	1		0.01				0.003	
Operator 2	0.86	2.37	[1.35, 4.18]	0.003	− 1.32	0.27	[0.12, 0.60]	0.001
Operator 3	0.62	1.87	[0.86, 4.07]	0.12	− 1.10	0.90	[0.40, 2.05]	0.81
Operator 4	0.67	1.96	[0.95, 4.07]	0.07	− 0.75	0.47	[0.19, 1.14]	0.10
Operator 5	1.22	3.38	[1.61, 7.11]	0.001	− 0.56	0.57	[0.25, 1.29]	0.18
Hancock	1		0.20				0.04	
SJR	0.56	1.75	[0.91, 3.38]	0.09	− 1.18	0.31	[0.10, 0.91]	0.03
ATS AP	0.79	2.20	[1.12, 4.32]	0.02	− 0.18	0.84	[0.30, 2.32]	0.73
CE magna	0.34	1.40	[0.82, 2.41]	0.22	− 0.37	0.69	[0.23, 2.10]	0.51
Others	0.37	1.44	[0.57, 3.69]	0.44	− 0.65	0.52	[0.20, 1.38]	0.19
Atrial fibrilation	− 0.03	0.97	[0.59, 1.60]	0.90				
AS dominant	0.05	1.05	[0.57, 1.91]	0.88				
Mitral regurgitation	0.43	1.54	[0.61, 3.85]	0.36				
CVA	− 0.03	0.97	[0.58, 1.61]	0.90				
Hypertension	0.04	1.05	[0.69, 1.58]	0.83				
PAD	0.70	2.01	[0.18, 22.37]	0.57				
Rheumatic disease	0.34	1.40	[0.68, 2.88]	0.36				
COPD	− 0.19	0.83	[0.25, 2.77]	0.76				
Diabetes mellitus	− 0.14	0.87	[0.54, 1.39]	0.55				
Heart failure	0.00	1.00	[0.42, 2.37]	1.00				
Malignancy	− 0.48	0.62	[0.32, 1.18]	0.15				
Dyslipidemia	0.11	1.12	[0.58, 2.16]	0.74				
CRF	− 0.24	0.79	[0.31, 2.06]	0.63				
History of MI	0.70	0.50	[0.37, 11.18]	0.42				
Liver cirrhosis	1.40	4.07	[0.45, 36.74]	0.21				
Ejection fraction	− 0.01	0.99	[0.98, 1.01]	0.56				
LVMI	0.001	1.00	[0.99, 1.01]	0.80				
Peak velocity, m/s	− 0.01	0.99	[0.80, 1.24]	0.94				
Mean PG, mmHg	0.00	1.00	[0.99, 1.01]	0.99				
Tricuspid valve				0.78				
Bicuspid without raphe	− 0.10	0.91	[0.53, 1.55]	0.72				
Bicuspid with raphe	0.12	1.13	[0.69, 1.84]	0.64				

AS, aortic stenosis; BNP, brain natriuretic peptide; BUN, blood urea nitrogen; CE magna, Carpentier-Edwards PERIMOUNT Magna; CI, confidence interval; COPD, chronic obstructive pulmonary diseases; CRF, chronic renal failure; CVA, cerebrovascular accident; eGFR, estimated glomerular filtration rate; LVMI, left ventricular mass index; MI, myocardial infarction; PAD, peripheral arterial disease; PG, pressure gradient; SJR, St Jude Medical Regent.

### Echocardiographic findings

The preoperative and postoperative echocardiographic data are shown in Supplementary Table 1. Preoperatively, left ventricular EF was significantly higher in group CT-Sim compared to group CT-Lg. LV ejection fraction was higher in group CT-Sim even in postoperative echocardiography, although LVMI and mean pressure gradient were higher in CT-Lg group. Peak velocity and pressure gradients were decreased compared to the preoperative values in both groups.

### Postoperative outcomes

Postoperative outcomes are noted in Supplementary Table 1. Postoperative paravalvular leakage, bleeding, cerebrovascular accident, postoperative kidney injury, admission due to cardiac problems, redo AVR, major adverse cardiac and cerebrovascular events (MACCE), death within 30 days after procedure, and overall mortality were not statistically different between CT-Lg and CT-Sim groups (*p* > 0.05).

## Discussion

In this study, bicuspid aortic valve parameters were well correlated with surgical aortic valve size as well as tricuspid valve parameters. Area-derived diameter showed the strongest correlation with TAD of surgical valves in patients with tricuspid or bicuspid valve without raphe, and perimeter-derived diameter showed highest correlation in patients with bicuspid valve with raphe. After propensity score matching and multivariable logistic regression analysis, operator factor and prosthetic valve types were remained as significant differences between intraoperative sizing and CT-based sizing.

In a recent study, intraoperative annular measurements in SAVR were compared using preoperative CT^9^, and perimeter-derived annular diameter was used instead of area-derived diameter based on their correlation results and previous literature^20–22^. However, they used nominated valve size, bicuspid aortic valve was excluded from these studies and the number of patients were relatively small. In this study, we evaluated CT-derived annular parameters using TAD of prosthetic valves which can reflect the real implanted valve size in a large cohort, including bicuspid aortic valve. Area-derived diameter showed strongest correlation among the CT-derived parameters in tricuspid and bicuspid valves without raphe groups. The results were consistent with Wang et al.’s study that also showed optimal measurement of annulus size in bicuspid aortic valve by area-derived diameter on CT. In the group of bicuspid valve with raphe, perimeter-derived diameter showed the strongest correlation with TAD. There are several recent studies dealing with the annular measurement on CT^23,24^. Boccalini et al. demonstrated that the annulus in bicuspid aortic valve undergoes significant change in shape during cardiac cycle similar to that encountered in tricuspid valves but with overall larger dimension^23^. The results was accord with the guidline suggesting to measure the aortic annulus in systolic phase^10^. Iannopollo et al. reported a novel supra-annular sizing method for TAVI, which measures the level of implantation at raphe plane perimeter to optimize prosthesis sizing in raphe-type bicuspid aortic valve disease. Although the number of patients in this study were small, TAVI based on this method showed successful results with no event of procedural complication. In our study, we did not consider this method, because in cases of SAVR, degeneration and calcification in native valve can be removed during the surgery and the prosthesis can be implanted in the virtual basal ring of aortic valve. However, in cases of TAVI, there are significant difference in situation considering the anatomy that native valve can not be removed while implanting the prosthesis, so this alternative method could be considered and it would be valuable to be validated in the future study.

In our study, 63.4% of patients had a valve smaller than that suggested by CT (group CT-Lg). Even though we modified the definition of CT-Lg using the difference between TAD and area-derived diameter which was used by previous studies^9^, the percentage of CT-Lg (69.7%) was higher than earlier reported. Another study of intraoperative echocardiography and CT images directly compared intraoperative annular sizing in 227 patients and showed that annular size on CT were larger in 72.2% of patients^25^. Various factors may affect the selection of implanted valve size in the operation field, such as physiologically arrested heart, aorta and left ventricular outflow shape, calcification burden at the annular level, and associated heart failure. Moreover, even if we used TAD of surgical valves, the annulus plane, which is determined by inserting the sizer in the surgical field and suturing the valve, is determined by the surgeon’s eyesight and experience; therefore, it may not be exactly at the annulus level confirmed by CT. In our study, operator factor and prosthetic valve types were remained as significantly associated factors to affect the difference between intraoperative sizing and CT-based sizing after multivariable logistic regression. This finding also suggest that the size of the intraoperative valve is highly dependent on the surgeon’s experience and judgement. The surgical techniques (i.e. supra-annular vs. intra-annular prosthesis placement, simple interrupted suture vs. pledgetted non-everting mattress suture technique) also can affect the size of the implanted prosthesis. In our center, supra-annular prosthesis placement was preferred because it can introduce larger prosthesis and obtain larger effective orifice area (EOA). Therefore, it could not be considered as a variable affecting the difference between CT-derived diameter and TAD. For the suture technique, which suture method is used depends on the operator, and the effect of suture technique is related and bounded to surgeon factors which is difficult to analyze them separately. The recent published study in our center showed that the surgeon-dependent factors are significant independent determinants for EOA in SAVR and the surgeons who showed a wider EOA used the simple interrupted suture rather than pledgetted non-everting mattress suture^26,27^.

Implanted SAVR were smaller relative to CT-based sizing, and the potential GOA was larger if patients had undergone transcatheter aortic valve replacement compared to the SAVR in same patients. GOA of the theoretical SAVR valves was based on Carpentier Edward Magna valves because Carpentier Edward Magna valves was the most commonly (32.3%) used valve in this study and is has an relatively larger orfice area compared with other types of prothestic valve. For similar reasons, we used SAPIEN 3 to compare GOA values (area-derived GOA) when the same patient received TAVR using SAPIEN 3 as a theoretical comparison because the SAPIEN 3 is most commonly used device for TAVR. The type of prosthetic valve used in this study for SAVR was actually very diverse, so to simplify the anlysis and compare the result easily between the cases of SAVR and theoretical TAVR, we decided to use the simulated value in patients with SAVR by converting to single valve type rather than using the actual value of diverse valve types from different manufactures. However, it could not reflect actual clinical scenario because clinical characteristics of TAVR candidates are still different from those with SAVR, even though the potential population for TAVR has been recently widened for low-risk patients. Since we coud not perform both SAVR and TAVR in the same patients, the evaluation for both groups are limited and caution is necessary for the interpretation of the results. Further studies should be performed for the evaluation of effect of GOA in TAVR and SAVR.

There were several limitations to this study. This study was retrospective, thus patients who required surgery could not represent all patients with aortic valve disease. However, considering that aortic stenosis is the most common aortic valvular disease and we include relatively large population cohort, the results from this study may be applicable for aortic annular sizing in general. Second, since the surgeon’s choice governs the types of prosthetic valves used, the “operator” factor may not be assessed as a factor, apart from the diversity of the valves. In our study, prosthetic valve types were varied. To obtain surgical reference standard from the diverse types of prosthetic valves, we used TAD as an indicator of all surgical valve sizes. Third, although we used intraoperative valve sizing, i.e., surgical prosthetic valve size, as a reference standard to compare CT annular sizing, intraoperative measurement could also be affected by the surgeon and the types of prosthetic valve used. Moreover, because native aortic valve, which can prevent expanding the TAVI device by mechanical force, remains and is crushed during the TAVI procedure, the annular sizing for surgical valve that requires suture fastening may not be directly applicable for TAVI. Fourth, recently, consensus statement has been published for the classification for bicuspid aortic valve. The classification includes fused, 2-sinus, and partial fusion type which recognizes the partial fusion type^28^. Our study analyzed the bicuspid aortic valve regarding the presence or absence of raphe following previously described classification by Sievers et al.^29^. However, little is known about whether the patients’ outcome, especially in case with SAVR or TAVR, is affected regarding the type of bicuspid aortic valve based on the new classification. Moreover, there is also no evidence that the different method for aortic annular measurement is needed for specific type of bicuspid aortic valve based on new classification. Finally, the clinical outcomes did not show significant assoiciation with the degree of discrepancies between CT-derived diameter and SAVR size. Howevere, the number of event was too small to conclude in our study (overall mortality, 6 [3.1%] in CT-Sim vs. 10 (2.4%) in CT-Lg). Thus, further studies are warranted.

In conclusion, bicuspid aortic annulus diameters measured on CT showed a significant correlation with surgical aortic valve size. Annular measurement methods for tricuspid valve could also be adjustable for bicuspid aortic valve sizing. The difference between CT-derived diameter and surgical AVR size is affected by operator factor and the types of prosthesis but not affected by the bicuspid valve.

## Supplementary Information

Supplementary Information.

## Abbreviations

AS: Aortic stenosis
BNP: Brain natriuretic peptide
CT: Computed tomography
EF: Ejection fraction
GOA: Geometric orifice area
LVMI: Left ventricular mass index
ICC: Intra-class correlation coefficient
SAVR: Surgical aortic valve replacement
TAD: Tissue annulus diameter
TAVR: Transcatheter aortic valve replacement
