# Supplementary material for: Aortic annulus sizing in bicuspid and tricuspid aortic valves using CT in patients with surgical aortic valve replacement
Source: Sci Rep. 2021 Oct 25;11:21005. doi: 10.1038/s41598-021-00406-3 (PMC8549004; doi:10.1038/s41598-021-00406-3)
Supplement: Supplementary file 1 — Supplementary Information. [file 41598_2021_406_MOESM1_ESM.docx]

**Supplementary material**

***CT protocol***

Cardiac CT scanning was performed using dual-source CT scanners (Somatom Force or Somatom Definition Flash, Siemens, Erlangen, Germany). Patients with no contraindication to beta blockers and with initial heart rates >75 beats/min received an oral dose of 2.5 mg bisoprolol (Concor, Merck, Darmstadt, Germany) 1 h before the CT examination. A bolus of 60–80 mL of nonionic, iodinated, intravenous contrast material (Iomeron, Bracco Imaging SpA, Milan, Italy) was injected using a power injector (Stellant D, Medrad, Indianola, PA, USA) at 4.0 mL/s. Following that, 40 mL of a 70:30 mixture of saline and contrast agent was injected. The scan delay was determined using the bolus-tracking method (region of interest, the ascending aorta; attenuation threshold level, 100 HU; scan delay, 8 s). A retrospective, ECG-gated spiral scan was performed, and ECG-based tube current modulation with a dose pulsing window of 0–90% of the R–R interval was applied. Tube voltage and tube current–exposure time product were adjusted according to the patients’ body size, and the scan parameters used were as follows: tube voltage, 80–120 kV; tube current–exposure time product, 185–380 mAs; collimation, 128 × 0.6 mm; and gantry rotation time, 280 s. The 3-dimensional CT data were reconstructed in a 10% R–R interval using a 1-mm slice and a B26 kernel, which were transmitted to a dedicated external workstation (Aquarius, TeraRecon, Foster City, CA) for post-processing.

**Supplementary Table 1. Tissue annulus diameter in different types of prosthetic vales used in the study arranged by labeled size**

| Valve type | Labeled size | TAD, mm | Valve type | Labeled size | TAD, mm |
| --- | --- | --- | --- | --- | --- |
| SJR | 19 | 19 | **Sorin Overline** | 18 | 17.2 |
|  | 21 | 21 |  | 20 | 19.2 |
|  | 23 | 23 |  | 22 | 21.3 |
|  | 25 | 25 | **CE magna** | 19 | 19 |
|  | 27 | 27 |  | 21 | 21 |
| ATS AP | 18 | 18.2 |  | 23 | 23 |
|  | 20 | 20.2 |  | 25 | 25 |
|  | 22 | 22.2 |  | 27 | 27 |
|  | 24 | 24.2 | **Hancock** | 21 | 21 |
|  | 26 | 26.2 |  | 23 | 23 |
| CM Top Hat | 19 | 14.7 |  | 25 | 25 |
|  | 21 | 16.7 |  | 27 | 27 |
|  | 23 | 18.5 | **Biocor** | 21 | 21 |
|  | 25 | 20.5 |  | 23 | 23 |
| Mitroflow | 23 | 23 | **Mosaic** | 19 | 19 |
|  | 25 | 25 |  | 23 | 23 |
| Trifecta | 21 | 21 | **On-X** | 23 | 21.4 |
|  | 23 | 23 |  | 25 | 23.4 |
|  | 25 | 25 |  | 27 | 25.4 |

TAD, tissue annulus diameter.

**Supplementary Table 2. CT measurements, postoperative echocardiographic findings, and postoperative outcomes regarding the groups based on discrepancy between diameter on CT from the surgical aortic valve size**

| **Variables** | CT-Sim  (n = 244) | CT- Lg  (n = 423) | *p* value^†^ |
| --- | --- | --- | --- |
| Implanted valve size | 23.0 ± 2.1 | 22.3 ± 2.3 | < 0.001 |
| Tissue annulus diameter | 23.0 ± 2.1 | 22.2 ± 2.5 | < 0.001 |
| GOA | 3.5 ± 0.7 | 3.3 ± 0.7 | 0.03 |
| **CT-derived parameters** |  |  |  |
| Maximal annulus diameter | 26.7 ± 2.7 | 29.5 ± 4.1 | < 0.001 |
| Minimal annulus diameter | 21.5 ± 2.2 | 24.2 ± 3.5 | < 0.001 |
| Mean annulus diameter | 24.1 ± 2.2 | 26.9 ± 3.6 | < 0.001 |
| Perimeter | 77.0 ± 6.9 | 85.5 ± 11.5 | < 0.001 |
| Perimeter-derived annulus diameter | 24.5 ± 2.2 | 27.2 ± 3.7 | < 0.001 |
| Area | 450.0 ± 83.3 | 565.9 ± 153.5 | < 0.001 |
| Area-derived annulus diameter | 23.8 ± 2.2 | 26.6 ± 3.5 | < 0.001 |
| Sinus diameter | 37.2 ± 5.3 | 40.0 ± 31.4 | 0.22 |
| ST junction diameter | 32.0 ± 5.6 | 32.1 ± 5.0 | 0.71 |
| Ascending aorta tubular portion | 41.0 ± 6.9 | 40.3 ± 6.3 | 0.13 |
| **Postoperative echocardiography** |  |  |  |
| EF | 58.9 ± 10.2 | 54.0 ± 13.4 | 0.01 |
| LVMI | 186.9 ± 68.6 | 224.9 ± 195.4 | 0.04 |
| Peak velocity | 2.6 ± 0.6 | 2.7 ± 0.6 | 0.12 |
| Mean pressure gradient | 14.7 ± 5.8 | 16.0 ± 6.9 | 0.98 |
| PVL, any grade | 20 (8.2) | 45 (10.6) | 0.31 |
| PVL > mild | 1 (0.4) | 3 (0.7) | 0.63 |
| **Patient-prosthesis mismatch** |  |  | < 0.001 |
| Normal | 54 (22.1) | 336 (79.4) |  |
| Mild/moderate | 48 (19.7) | 85 (20.1) |  |
| Severe | 0 (0) | 1 (0.2) |  |
| **Postoperative complications** |  |  |  |
| Postop. bleeding | 9 (3.7) | 16 (3.8) | 0.95 |
| Early CVA | 2 (0.8) | 2 (0.5) | 0.63 |
| Infection | 3 (1.2) | 7 (1.7) | 0.61 |
| Dialysis | 11 (4.5) | 22 (5.2) | 0.69 |
| ECMO use | 4 (1.6) | 6 (1.4) | 0.84 |
| CVA | 5 (2.0) | 6 (1.4) | 0.55 |
| Cerebral infarction | 3 (1.2) | 5 (1.2) | > 0.99 |
| Cerebral hemorrhage | 2 (0.8) | 1 (0.2) | 0.25 |
| Admission due to heart failure | 2 (0.8) | 4 (0.9) | 0.89 |
| due to bleeding | 1 (0.4) | 1 (0.2) | 0.63 |
| due to arrhythmia | 1 (0.4) | 5 (1.2) | 0.29 |
| due to IHD | 2 (0.8) | 4 (0.9) | 0.89 |
| due to angina | 1 (0.4) | 3 (0.7) | 0.63 |
| due to MI | 1 (0.4) | 1 (0.2) | 0.63 |
| MACCE | 11 (4.5) | 19 (4.5) | > 0.99 |
| Redo AVR | 2 (0.8) | 2 (0.5) | 0.63 |
| Early mortality | 5 (2.0) | 7 (1.7) | 0.78 |
| Overall mortality | 6 (2.5) | 10 (2.4) | 0.94 |

Note.−Data are shown as the number of patients with percentages in parentheses or mean ± standard deviation.

%Difference = (Area-derived annulus diameter on CT – TAD) / TAD × 100. Each group classified as follows. CT-Sim: %Difference ≤ 10 and CT-Lg: %Difference > 10.

CVA, cerebrovascular accident; MI, history of myocardial infarction; LV, left ventricle; ECMO, extracorporeal membrane oxygenation; PVL, paravalvular leakage; IHD, ischemic heart disease; MACCE, major adverse cardiac and cerebrovascular events; AVR, aortic valve replacement.

**Supplementary Table 3. Interobserver agreements for CT parameters**

| CT parameters | Intraclass correlation coefficient (*p* value) |
| --- | --- |
| Annular maximal diameter | 95.9 (< 0.001) |
| Annular maximal diameter | 93.9 (< 0.001) |
| Annular perimeter | 96.7 (< 0.001) |
| Annular area | 96.1 (< 0.001) |
| Sinus of Valsalva maximal diameter | 97.0 (< 0.001) |
| Sinotubular junction diameter | 89.2 (< 0.001) |
| Ascending aorta tubular portion | 98.6 (< 0.001) |

**Supplementary Figure 1. Box-and-whiskers plot displaying geometric orifice area for tricuspid, bicuspid with raphe and bicuspid without raphe groups** (*p < 0.001 vs. SAVR, # p < 0.001 vs. SAVR-CT). GOA, geometric orifice area; SAVR, surgical aortic valve replacement; and TAVR, transcatheter aortic valve replacement.


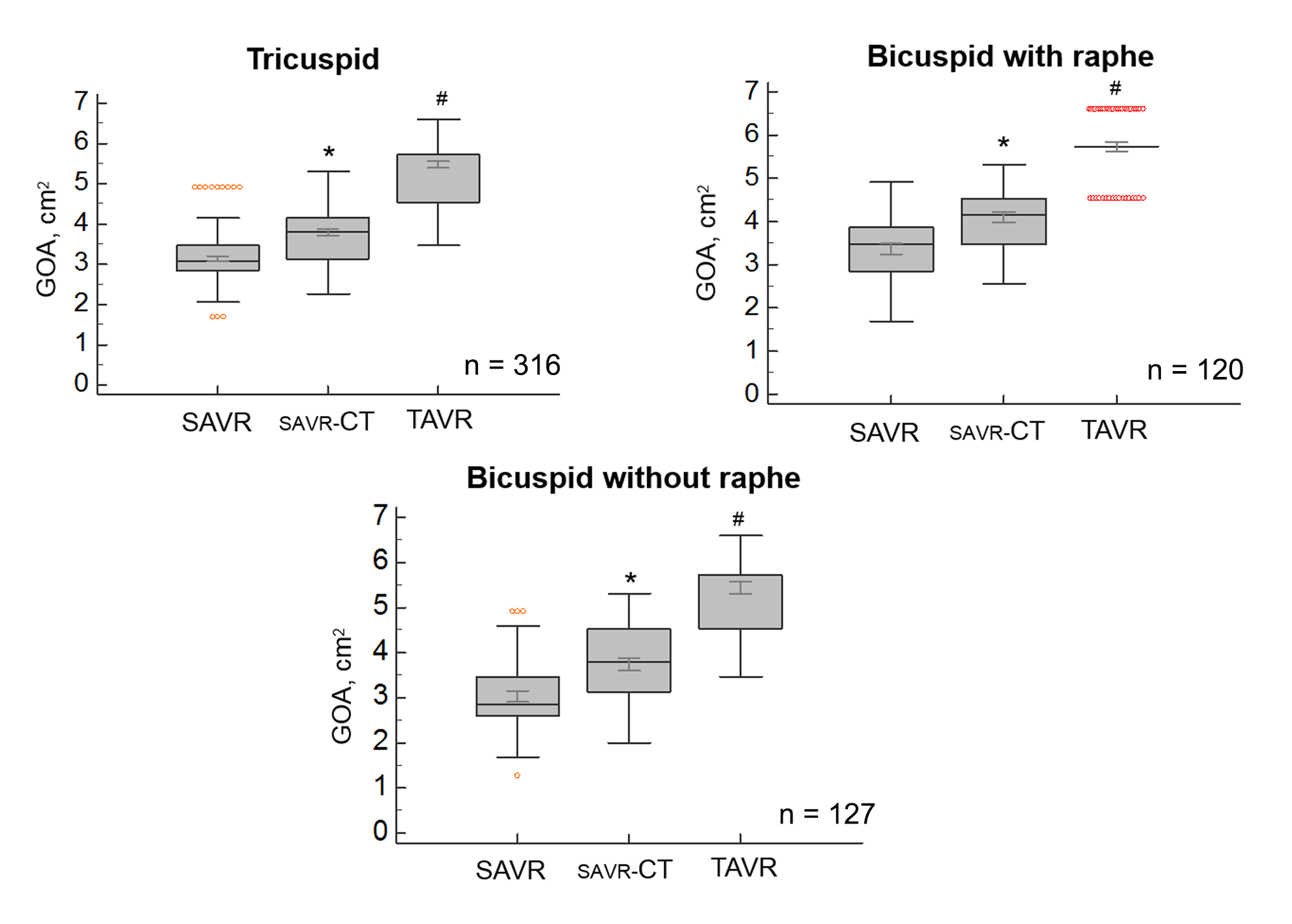


**Supplementary Figure 2. Area-derived annular diameter on CT compared to the surgically implanted valve size**
